# Development of Zn-CoS@Ni(OH)_2_ Heterostructured Nanosheets for High-Performance Supercapacitors

**DOI:** 10.3390/molecules29246022

**Published:** 2024-12-20

**Authors:** Hengxu Cheng, Jian Wang, Shiwei Song, Meizhen Dai, Yucai Li, Dong Zhang, Depeng Zhao

**Affiliations:** School of New Energy, Shenyang Institute of Engineering, Shenyang 110136, China

**Keywords:** nickel-cobalt-based sulfides, supercapacitors, Zn-CoS@Ni(OH)_2_

## Abstract

With the increasing societal demand for sustainable and renewable energy, supercapacitors have become research hotspots. Transition metal oxides, due to their high capacitance and abundant resources, are the preferred electrode materials. However, their poor conductivity and volume changes limit performance enhancement. Therefore, the development of heterogeneous structure electrode materials has become an important research direction. In this study, Zn-CoS@Ni(OH)_2_-1 nanosheets were synthesized on a nickel foam substrate via a three-step hydrothermal synthesis method, exhibiting excellent capacitance performance. In terms of capacitance, the material achieved a specific capacitance of 624 F/g at a current density of 1 A/g. When assembled into an asymmetric supercapacitor with Active Carbon materials, the device demonstrated an energy density of 35.4 Wh kg^−1^.

## 1. Introduction

With the continuous advancement of society and significant improvements in living standards, the demand for sustainable and renewable energy has become increasingly urgent [1]. In the pursuit of efficient electrochemical energy storage solutions, supercapacitors have emerged as a prominent research field due to their outstanding fast charge–discharge capability, remarkable power density, and environmentally friendly characteristics [2,3]. Transition metal oxides have become the preferred electrode materials due to their relatively high specific capacitance and abundant resource availability [4,5,6]. However, these materials suffer from poor conductivity and are prone to volume changes during charge–discharge cycles, which limits the enhancement of their electrochemical performance. Therefore, developing heterogeneous structure electrode materials with large surface areas and rich active sites has become an important research direction to improve supercapacitor performance.

In recent decades, extensive research has highlighted the potential of transition metal-based nanomaterials in supercapacitors and electrocatalysis, especially considering their low cost, abundant availability, and excellent electrochemical performance [7,8,9]. These nanomaterials come in various forms, including oxides, sulfides, phosphides, and nitrides. Transition metal sulfides have gained significant attention due to their outstanding electrocatalytic performance and superior catalytic stability [10,11,12]. However, in recent years, research progress using single metal oxides (such as Co_3_O_4_, CuO, and NiO) for supercapacitors has plateaued [13]. Therefore, researchers have increasingly shifted their focus to mixed transition metal oxides containing multiple metal elements. This shift is attributed to the advantages of these mixed oxides, including higher capacitance, lower resistance, the promotion of reversible redox reactions through multiple oxidation states, and synergistic effects between different metals [14,15,16]. Furthermore, an effective strategy to enhance electrocatalytic performance is the sulfuration of these oxides. Current studies show that ternary metal sulfides generally exhibit more universal redox reactions and higher conductivity compared to their corresponding single-metal sulfides [17]. As a result, the research on ternary mixed metal sulfides has been growing. For example, Yu et al. reported NixCo_3−x_S_4_ tetrahedral hollow nanocapsules for supercapacitors. The innovative hollow pyramidal structure exhibited excellent capacitance performance, achieving a specific capacitance of 757.4 F/g at a current density of 5 A/g, and 585.2 F/g at 20 A/g [18].

In this study, Zn-CoS@Ni(OH)_2_-1 nanosheets were synthesized on a nickel foam substrate via a simple, convenient, and easy-to-operate three-step hydrothermal method. Compared to the ternary metal sulfide CoS@Ni(OH)_2_ alone, the doping of Zn elements led to synergistic effects between Zn and Co, Ni, and other elements, resulting in improved conductivity, higher specific capacitance, and greater electrochemical activity for Zn-CoS@Ni(OH)_2_-1. The material exhibited excellent capacitance performance, with a specific capacitance of 624 F/g at a current density of 1 A/g. When assembled into an asymmetric supercapacitor with Active Carbon materials, the device demonstrated an energy density of 35.4 Wh kg^−1^.

## 2. Results and Discussion

**Figure 1:**
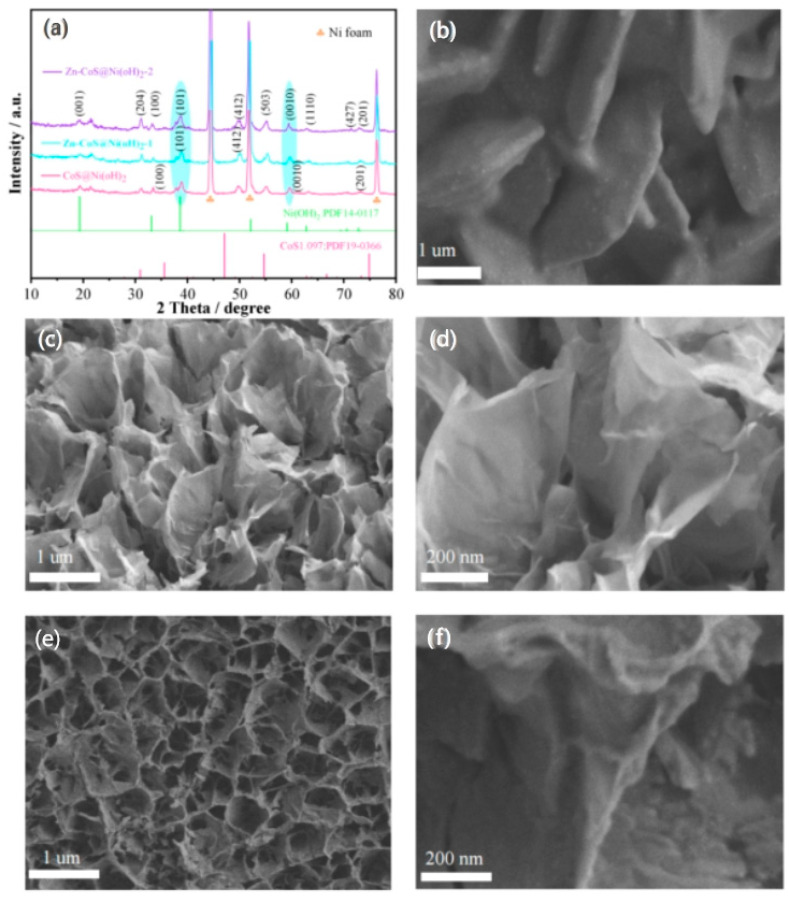
(**a**) XRD of samples; (**b**) SEM images of CoS@Ni(OH)_2_ samples; (**c**,**d**) SEM images of Zn-CoS@Ni(OH)_2_-1 samples; (**e**,**f**) SEM images of Zn-CoS@Ni(OH)_2_-2 samples.

To investigate the effects of varying Zn ion doping concentrations on the material, X-ray diffraction (XRD) and scanning electron microscopy (SEM) were employed. Figure 1a shows the XRD patterns of the samples with different doping concentrations, revealing the crystal structure and composition. Strong diffraction peaks at 44.28°, 51.8°, and 76.32° correspond to the characteristic peaks of nickel foam. Peaks at 19.26°, 33.06°, 38.54°, and 72.74° correspond to the (001), (100), (101), and (201) planes of Ni(OH)_2_ (PDF#14-0117). Peaks at 30.91°, 49.10°, 55.48°, 59.56°, 62.77°, and 71.28° correspond to the (204), (412), (503), (0010), (1110), and (427) planes of CoS_1.097_ (PDF#19-0366). These results confirm the successful coating and doping of Zn ions into the CoS@Ni(OH)_2_ nanosheets. Moreover, Zn-CoS@Ni(OH)_2_-1 and Zn-CoS@Ni(OH)_2_-2 show clear shifts in diffraction peaks at 38.54° and 59.56°, indicating that Zn doping has altered the original crystal structure. Figure 1b shows the SEM image of CoS@Ni(OH)_2_ nanosheets, which reveals the material’s regular nanosheet structure. Figure 1c,d shows SEM images of Zn-CoS@Ni(OH)_2_-1 nanosheets doped with 0.15 mM Zn^2+^, exhibiting significant structural changes and the formation of a nanoflower structure. The introduction of Zn^2+^ induces structural shifts that create more electrochemically active surface area. Figure 1e,f shows the SEM images of Zn-CoS@Ni(OH)_2_-2 nanosheets doped with 0.3 mM Zn^2+^, where increasing Zn concentration leads to more vacancies and a transformation from nanosheet to nanoflower structures [19,20].

**Figure 2:**
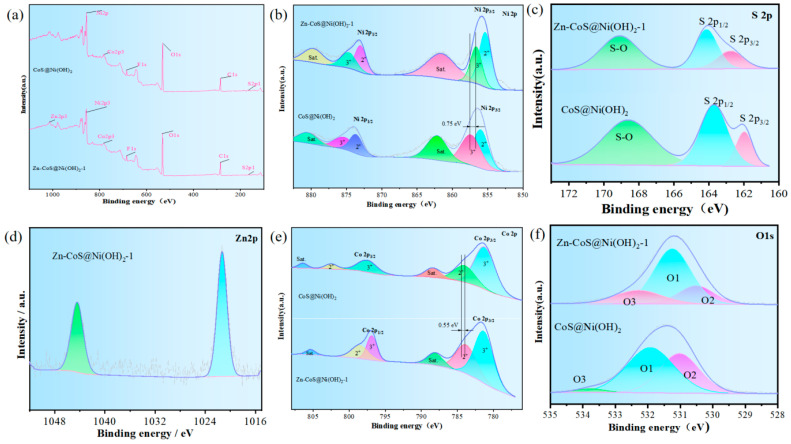
The structural features of the electrocatalysts of the prepared materials. (**a**) XPS full spectrum of the sample; (**b**) Ni 2p; (**c**) S 2p; (**d**) Zn 2p; (**e**) Co 2p; (**f**) O 1s.

To further investigate the elemental composition and oxidation states, XPS analysis was performed. Figure 2a compares the full spectra and elemental composition of CoS@Ni(OH)_2_ and Zn-CoS@Ni(OH)_2_-1, confirming the presence of Zn, Ni, Co, O, and S elements in the latter, thus validating the successful doping of Zn. Figure 2b presents the Ni 2p spectra of CoS@Ni(OH)_2_ and Zn-CoS@Ni(OH)_2_-1, showing peaks indicative of Ni^2+^ and Ni^3+^ species, with a 0.75 eV binding energy difference for Ni^3+^ after Zn doping [21]. Figure 2c shows the S 2p spectra, indicating sulfur–metal bonding, low-coordination sulfur ions, and surface oxidation states. Figure 2d presents the Zn 2p spectra of Zn-CoS@Ni(OH)_2_-1, showing the characteristic Zn 2p peaks at 1021.28 eV and 1044.28 eV [22,23]. Figure 2e shows the Co 2p spectra, revealing shifts in binding energies for Co^2+^ and Co^3+^ after Zn doping [24]. Figure 2f compares the O 1s spectra for different doping concentrations, showing a shift to lower binding energies after Zn doping and a blue shift in the O 1s spectra [25].

**Figure 3:**
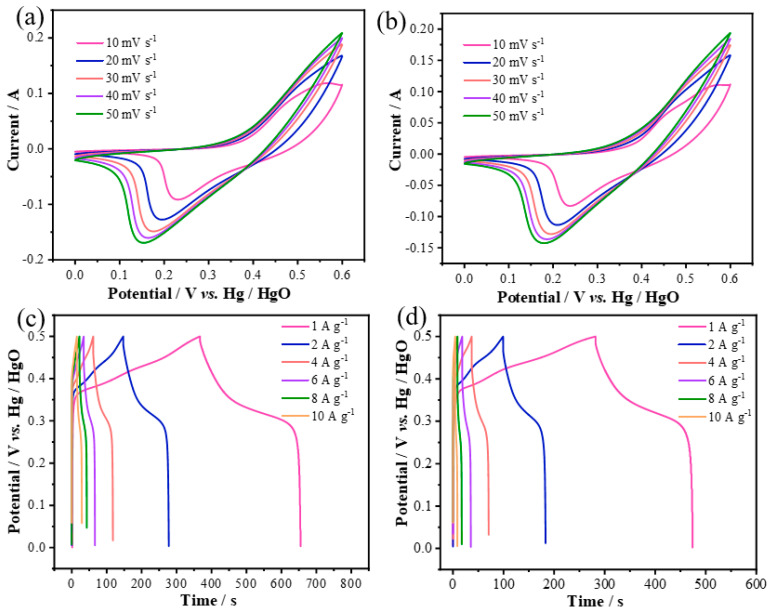
Superelectrical properties of CoS@Ni(OH)_2_ and Zn−CoS@Ni(OH)_2_−2 samples. (**a**) CV curves of CoS@Ni(OH)_2_ at different scanning speeds; (**b**) CV curves of Zn-CoS@Ni(OH)_2_−2 at different scanning speeds; (**c**) CP curves of CoS@Ni(OH)_2_ at different current densities; (**d**) CP curves of Zn−CoS@ CP curves at different current densities for Ni(OH)_2_−2.

Figure 3a shows the CV curve of CoS@Ni(OH)_2_ nanosheet electrodes at different scan rates, revealing oxidation/reduction peaks that shift linearly without significant polarization, indicating good stability. Figure 3b shows the CV curve for Zn-CoS@Ni(OH)_2_-2, exhibiting similar characteristics but with a smaller enclosed area due to the higher doping concentration. Figure 3c shows the GCD charge–discharge curves of CoS@Ni(OH)_2_, with a large voltage platform, indicating good reversibility and effective charge transfer. At current densities of 1, 2, 4, 6, 8, and 10 A g^−1^, the discharge times were 288, 263.8, 230.8, 199.8, 168, and 144 s, respectively, corresponding to specific capacitances of 576, 527.6, 461.6, 399.6, 336, and 288 F/g. The electrode maintained 50% capacity retention, demonstrating excellent rate capability. Figure 3d shows the GCD curves of Zn-CoS@Ni(OH)_2_-2, which exhibit similar characteristics but with shorter discharge times and lower specific capacitances of 388, 346.4, 275.2, 211.2, 139.2, and 86 F/g at current densities of 1–10 A g^−1^, reflecting a lower capacity retention rate of 22% due to the higher doping concentration.

**Figure 4:**
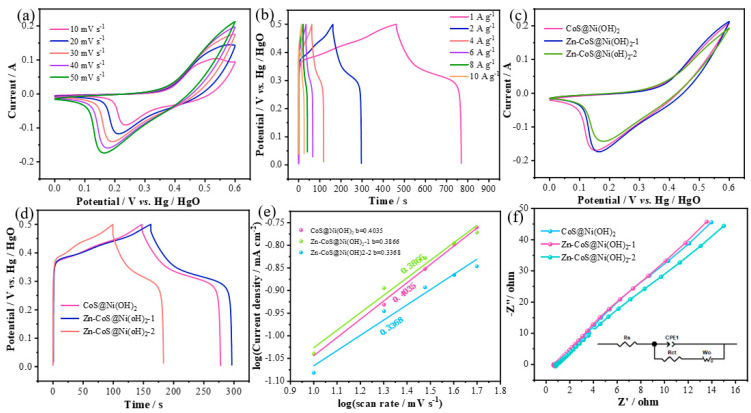
Comparison of the superlattice properties of Zn-CoS@Ni(OH)_2_−1 samples and the superlattice properties of all the samples; (**a**) CV curves of Zn-CoS@Ni(OH)_2_-1 at different scanning speeds; (**b**) CP curves of Zn-CoS@Ni(OH)_2_−1 at different scanning speeds; (**c**) CV curves of Zn-CoS@Ni(OH)_2_−1 at same scanning speeds; (**d**) CP curves of Zn-CoS@Ni(OH)_2_−1 at the same current density; (**e**) peak current plot (**f**) Nyquist plot.

Figure 4a shows the CV curves of Zn-CoS@Ni(OH)_2_-1 nanosheets at different scan rates. It can clearly be seen that the CV characteristics of this material are similar to those of the two electrode materials. Figure 4b presents the GCD curves of Zn-CoS@Ni(OH)2-1 nanosheets at different current densities. The GCD curve characteristics of this material are similar to those of the two materials in Figure 1. The discharge times at current densities of 1–10 A g^−1^ are 312, 134.4, 58.7, 32.5, 20.1, and 12.9 s, respectively. From these, the specific capacitance values at different current densities are calculated to be 624, 537.6, 469.6, 390, 321.6, and 258 F/g. To further investigate the electrochemical performance of CoS@Ni(OH)_2_ nanosheets doped with different concentrations of Zn ions, we compared them, as shown in Figure 4c, which compares the CV curves of the nanosheets doped with various Zn concentrations at a scan rate of 50 mV/s. It is evident that Zn-CoS@Ni(OH)_2_-1 nanosheets have the largest enclosed area and the highest peak. Figure 4d shows the GCD curves of the nanosheets doped with different Zn concentrations at a current density of 2 A g^−1^. The discharge times for these materials at this current density are 131.9, 134.4, and 86.6 s, respectively, resulting in specific capacitances of 527.6, 537.6, and 346.4 F/g. It can be observed that as the Zn ion concentration increases, the discharge times initially increase and then decrease. This trend is more pronounced in the specific capacitance. For a more accurate analysis of their electrochemical reaction kinetics, we analyzed the peak current at the lowest point of the redox peaks in the CV curves at different scan rates, and constructed a peak current graph to evaluate the reaction type. According to the capacitance contribution formula [26,27,28]:

$$i=av^{b}$$

where i is the peak current at the lowest point, a and b are constants, and v is the scan rate. The b-value can be obtained by calculating the slope of the log(i) vs. log(v) plot, as shown in Figure 4e. When b is between 0 and 0.5, the reaction is primarily controlled by surface processes, while when b is between 0.5 and 1, the reaction gradually shifts from surface-controlled to diffusion-controlled. From the figure, it can be seen that the b-values for CoS@Ni(OH)_2_, Zn-CoS@Ni(OH)_2_-1, and Zn-Co@Ni(OH)_2_-2 nanosheets are 0.4035, 0.3866, and 0.3368, respectively. Therefore, the energy storage mechanism of these three materials is mainly controlled by surface reactions. To further evaluate the electrochemical reaction kinetics of these materials, EIS was employed, as shown in Figure 4f, where the x-axis and y-axis represent the real and imaginary axes, respectively. From the figure, it can be observed that the effective resistances of CoS@Ni(OH)_2_, Zn-CoS@Ni(OH)_2_-1, and Zn-Co@Ni(OH)_2_-2 nanosheets are 0.7406, 0.6544, and 0.8122 Ω, respectively. Therefore, Zn-CoS@Ni(OH)_2_-1 nanosheets exhibit the smallest effective resistance during the entire chemical reaction process, leading to the fastest surface charge transfer rate. Furthermore, the small radius of the high-frequency arc and the large slope of the low-frequency straight line indicate excellent capacitive properties [29,30].

**Figure 5:**
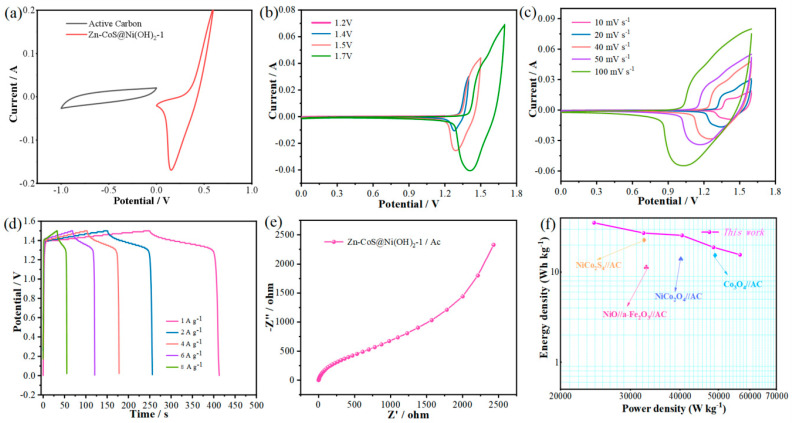
Performance of Zn-CoS@Ni(OH)_2_−1//AC asymmetric supercapacitor; (**a**) operating voltage of the capacitor; (**b**) CV curves at different voltages; (**c**) CV curves at different scanning speeds; (**d**) CP curves at different current densities; (**e**) Nyquist plots; (**f**) energy density plots.

A series of electrochemical performance analyses of CoS@Ni(OH)_2_, Zn-CoS@Ni(OH)_2_-1, and Zn-Co@Ni(OH)_2_-2 nanosheets revealed that Zn-CoS@Ni(OH)_2_-1 nanosheets outperform the other materials. To further investigate the practical application of this material, an asymmetric supercapacitor was assembled using Active Carbon as the cathode and Zn-CoS@Ni(OH)_2_-1 nanosheets as the anode. To explore the optimal voltage window for the supercapacitor, CV curves of the Active Carbon and Zn-CoS@Ni(OH)_2_-1 nanosheet electrodes were measured in a three-electrode system with a 3 M KOH electrolyte, as shown in Figure 5a. It is observed that the voltage window of Active Carbon is −1 to 0 V, while the voltage window of Zn-CoS@Ni(OH)_2_-1 nanosheets is 0 to 0.6 V, indicating that the maximum stable voltage for the supercapacitor is 1.6 V. Figure 5b shows the CV curves of the asymmetric supercapacitor at 1.2 to 1.7 V with a scan rate of 50 mV/s. As the voltage increases, the area of the curve also increases, but the shape remains almost unchanged, demonstrating good rate performance. Figure 5c presents the CV curves at the same voltage but with scan rates ranging from 10 to 100 mV/s. The shape of these curves is similar to that of the Zn-CoS@Ni(OH)2-1 nanosheet electrode, with redox peaks appearing similarly, suggesting that the device exhibits classic pseudocapacitive behavior due to fast, reversible surface redox reactions. As the scan rate increases, the peak values increase linearly, with minimal polarization, and the area enclosed by the curve also increases while maintaining its shape. This indicates that the Active Carbon//Zn-CoS@Ni(OH)_2_-1 asymmetric supercapacitor exhibits excellent stability. Figure 5d shows the GCD curves of the device at different current densities, revealing perfect symmetry. This suggests that the device exhibits superior reversibility, enabling fast charge transfer and efficient electrochemical oxidation or reduction reactions. The discharge times at current densities of 1, 2, 4, 6, and 8 A g^−1^ are 162, 106, 75, 52, and 25 s, respectively, result in specific capacitances of 324, 424, 600, 624, and 400 F g^−1^ at these current densities. To further investigate the electrochemical performance, an EIS analysis of the device was performed, as shown in Figure 5e. The effective resistance of the Active Carbon//Zn-CoS@Ni(OH)_2_-1 asymmetric supercapacitor was found to be 0.622 Ω, indicating excellent capacitive performance. The small radius of the high-frequency arc and large slope of the low-frequency straight line further demonstrate good capacitive characteristics [31,32]. To assess the practical application value of the device, energy density and power density curves were obtained from the GCD curves. Figure 5f shows the Ragone plot of the Active Carbon//Zn-CoS@Ni(OH)_2_-1 asymmetric supercapacitor, where the device achieves an energy density of 35.4 Wh kg^−1^ at a power density of 24,300 W kg^−1^, outperforming other previously assembled asymmetric supercapacitors such as NiCo_2_O_4_//AC (25.5 Wh kg^−1^), Co_3_O_4_//AC (17.9 Wh kg^−1^), and NiCo_2_O_4_//AC (15.42 Wh kg^−1^).

## 3. Experimental Section

### 3.1. Material Preparation

Cobalt (II) nitrate hexahydrate, nickel (II) nitrate hexahydrate, anhydrous sodium sulfide, urea, ammonium fluoride, potassium hydroxide, anhydrous ethanol, perchloric acid (HCl), Pt/C, and nickel foam were purchased from Sigma-Aldrich Chemical Company. All chemicals and materials were of analytical grade and used without further purification. In this experiment, all aqueous solutions were prepared using deionized water.

Nickel foam was first cleaned six times using an ultrasonic cleaner, with 30 min of alcohol cleaning followed by 30 min of deionized water washing. This process was repeated three times. After cleaning, the foam was dried at 60 °C for 24 h. A solution was prepared by dissolving 1.5 mM Co(NO_3_)_2_·6H_2_O, 6 mM NH_4_F, and 8 mM urea, in 60 mL deionized water. A specific amount of Zn(NO_3_)_2_·6H_2_O was added to this solution. The pre-treated nickel foam was placed into an autoclave and reacted at 120 °C for 6 h. After the reaction, the foam was allowed to cool to room temperature, then extracted and washed multiple times with deionized water. The foam was dried at 60 °C for 6 h. A sulfuration treatment was then carried out by dissolving 0.35 g Na_2_S·9H_2_O in 60 mL deionized water and stirring for 45 min. The precursor was added to an autoclave and reacted at 120 °C for 4 h, followed by natural cooling to room temperature, washing, and drying.

### 3.2. Material Characterization

Morphology and crystal structure of the as-prepared products were characterized through X-ray power diffraction (XRD, Shimadzu-7000, Cu Kα), X-ray photoelectron spectroscopy (XPS, ESCALAB 250 with an Al Kα), and scanning electron microscopy (SEM, Gemini 300-71-31).

### 3.3. Electrochemical Testing

Electrochemical performance was tested using a three-electrode system with a CHI 660E electrochemical workstation in 3 M KOH and 1 M KOH solutions. The as-prepared samples served as the working electrode, platinum (Pt) foil was used as the counter electrode, and saturated Hg/HgO and Ag/AgCl electrodes were used as reference electrodes. The variety of techniques used includes cyclic voltammetry (CV), constant current charge–discharge (CP), electrochemical impedance spectroscopy (AC), electrocatalysis, and chronopotentiometry.

## 4. Conclusions

In this study, a simple three-step hydrothermal method was used to successfully prepare the Zn-CoS@Ni(OH)_2_-1 catalyst. Compared to the ternary metal sulfide CoS@Ni(OH)_2_, the doping of Zn induces synergistic effects between Zn and other elements such as Co and Ni. The crystal structure of Zn-CoS@Ni(OH)_2_-1 exhibits enhanced conductivity, higher specific capacitance, and improved electrochemical activity compared to CoS@Ni(OH)_2_. In terms of supercapacitor performance, at a current density of 1 A/g, the material exhibits a discharge time of 312 s and a specific capacitance of 624 F/g. When assembled into an asymmetric supercapacitor with Active Carbon, the device achieves an energy density of 35.4 Wh/kg and a power density of 24,300 W/kg, demonstrating excellent capacitive performance. 

## Data Availability

Data will be made available on request.
